# Communicating the utility of the microbiome and bioinformatics to small flock poultry producers

**DOI:** 10.1016/j.psj.2022.101788

**Published:** 2022-02-16

**Authors:** Steven C. Ricke, Dana K. Dittoe, Ashley A. Tarcin, Michael J. Rothrock Jr.

**Affiliations:** ⁎ Meat Science and Animal Biologics Discovery Program, Department of Animal and Dairy Sciences, University of Wisconsin, Madison, WI 53706, USA; † USDA-ARS, U.S. National Poultry Research Center, Egg Safety & Quality Research Unit, Athens, GA 30605, USA

**Keywords:** bioinformatics, omics, microbiome, small poultry growers, education

## Abstract

The use of “omics” has become widespread across poultry production, from breeding to management to bird health to food safety and everywhere in between.  While the conventional poultry industry has become more exposed to the power and utility of “omic” technologies, smaller poultry flock producers typically do not have this same level of experience. Because smaller, nonconventional poultry production is a growing portion of the overall poultry market, it is important that they also have educational access to these research tools and the resultant data. While small flock producers are dedicated and knowledgeable farmers, their knowledge of these newer technologies may be limited at best, and it is the task of academic researchers to communicate the importance of these “omic” tools and how the omic data can improve a variety of different aspects of their operations. This review discusses ways to effectively communicate complex microbiota and microbial genome sequence data to small flock producers and transforming this data into meaningful and applicable information that they can utilize to inform beneficial management decisions.

## INTRODUCTION

Non-conventionally raised food animals for meat and egg production that are linked economically to local markets continues to grow in popularity (Stanton et al., 2018; Ricke and Rothrock, Jr., 2020). There have been several reasons suggested for this upswing in popularity. In general, this type of agricultural practice fits with the trend of sustainable agriculture becoming a consumer preference in the United States (Yue et al., 2020). Along these lines, interest has also increased toward antibiotic-free broiler meat based on consumer perceptions that are becoming more mainstream internationally (Haque et al., 2020). However, many issues critical to conventional poultry production such as food safety, nutrition, meat quality, and processing are also important concerns in pasture-raised poultry. In addition, for some nonconventional products such as organic eggs, price is an important factor to the consumer in some countries (Yeh et al., 2020). As local retail markets for pasture-raised poultry have become more prominent, further research to develop management tools and to better understand environmental issues have been conducted (Rothrock, Jr. et al., 2019a). As several surveys of pasture flock growers have indicated, management practices are quite variable among individual producers (Elkhoraibi et al., 2014; 2017; Ricke and Rothrock, Jr. 2020). There are likely several contributing factors to this variability in management including seasonal and geographical differences as well as a wide range of poultry breed, nutritional, and health practices (Jeni et al., 2021a,b). Although not well-documented, turnover of producers entering and leaving pasture flock production may also contribute to the observed variation in management practices because some of the incoming producers are novices at poultry husbandry practices.

Pasture flock poultry production remains challenging, not only because birds are raised under environmentally demanding conditions, but processing accessibility is still problematic despite the development of mobile processing units (O'Bryan et al., 2014; Mancinelli et al., 2018; Ricke and Rothrock, Jr., 2020). Shifting market demands also unpredictably impact production and processing logistics, while other concerns such as ensuring food safety also remain unclear. Numerous studies have examined the incidence of foodborne pathogens and implications for food safety in pasture flock poultry. However, these studies were primarily focused on isolated experimental or commercial settings and did not offer opportunities to provide more universal recommendations (Golden et al., 2021). Furthermore, potential sources of foodborne pathogens and subsequent contamination of pasture flock birds during production and processing are complex and it remains difficult to identify all potential exposure opportunities.

**Figure 1:**
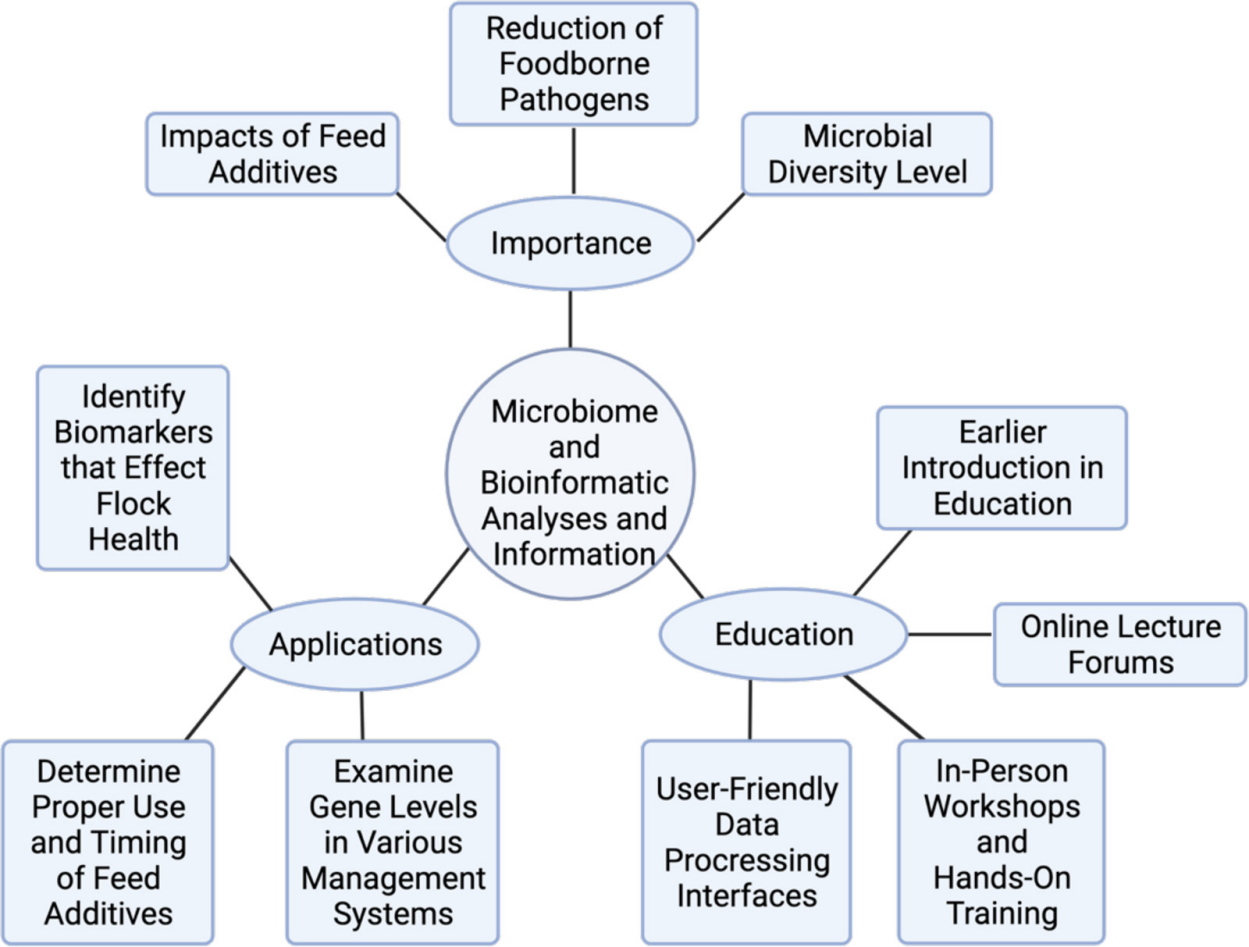
Importance and Utilization of Microbiome and Bioinformatic Data and Analyses. As microbial and bioinformatic assays have become more widely employed throughout poultry production, small poultry remain unaware of the benefits of these techniques. Illustrating the importance, demonstrating the applications, and providing education of these analyses serves as avenues to further engage small poultry producers in these practices. Figure created with Biorender.com.

There is a clear need to optimize the health and performance of birds raised under pasture conditions (Jeni et al., 2021b). Consequently, numerous feed additives have been suggested and to some extent implemented for pasture flock poultry production. These include both prebiotics and probiotics which have been the subject of several reviews (Ricke, 2015b, 2021a; Jeni et al., 2021a). To determine the efficacy of potential pathogen reducing feed additives, the mode of action within the gastrointestinal tract (**GIT**) of the bird must be understood. Recent years have seen the introduction of microbiome 16S rDNA-based analyses which has produced a new level of interpretable results for formulating some general observations. However, communicating the utility of these approaches and explaining how data can be incorporated into practical management practices to the lay producer audience remains challenging. In this review, the current understanding of the pasture flock microbiome will be discussed along with the practical importance of this type of information and potential communication strategies for use with producers (Figure 1).

## MICROBIOME APPLICATIONS FOR POULTRY PRODUCTION: RATIONALE AND CURRENT CONCEPTS

A key criterion for introduction of any new technological tools in agricultural operations is justification of the need for their respective applications, which can provide an economic advantage during the growth cycle of the animal. In conventional poultry production this can be in the form of more efficient feed management such as better feed conversion, and/or improved growth performance for broilers or egg production in egg layer operations. Optimizing feed management has become an especially more critical issue when conventional corn and soy sources become more costly (Cowieson and Kluenter. 2019). Consequently, feed additives such as feed enzymes have been introduced in an attempt to improve digestibility, overall utilization of feed conversion as well as other benefits (Cowieson and Kluenter. 2019). Probiotic cultures have also been screened for exogenous enzyme production (Danilova and Sharipova. 2020).

Minimizing foodborne pathogen occurrence in poultry broiler and laying hen operations is a primary issue for the poultry industry (Ricke, 2017; 2021b). While the economic advantages for feed additives to improve food safety in poultry production may not be as clear cut as performance attributes, improving food safety at all levels from the farm through processing and retail is important from a public health and governmental regulatory standpoint. Not surprisingly a wide range of feed additives have been developed and commercially introduced to the poultry industry which possess properties that either eliminate foodborne pathogens already present in the GIT and/or prevent their ability to colonize the GIT (Nisbet, 2002; Joerger, 2003; Patterson and Burkholder, 2003; Ricke, 2003, Ricke, 2018; Hajati and Rezaei, 2010; Hume, 2011; Clavijo and Flórez, 2018; Dittoe et al., 2018; Teng and Kim, 2018). It has become clearer that introduction of feed amendments into the GIT can elicit both direct and indirect influences on the GIT microbiota. Historically, GIT microbial ecology was based on microbial culturing, isolation and identification of specific members of the GIT microbial community (Ricke, 2015a). However, advancements in molecular methodology have greatly accelerated the ability to characterize the GIT microbial communities more completely (Ricke et al., 2017).

Like any technological development, the field of microbiome analyses has evolved its own specific set of terms and definitions. Much of this development and incorporation into food production systems has been the result of rapid advances in sequencing technology as well as the bioinformatic computer programs created to analyze the raw sequencing data (Ricke et al., 2017). Fundamentally, 2 types of microbial ecology information are sought with bioinformatic analyses. Certainly, taxonomic identification of the individual members of a given microbial community is possible based on sequencing of selected variable regions of the 16S rDNA gene. Although not quantitative, taxonomic profiles generated from 16S rDNA microbiome sequencing do provide a sense of the relative proportions of the respective taxonomic groups within the microbial consortia. Since sequence databases are incomplete, species identification can be incomplete, particularly at the species taxonomic level and distinct microbiome sequences are identified simply as Operational Taxonomic Units (**OTU**s). Once the taxonomic profile of a particular set of samples is complete, these can be presented graphically as numerical proportions within a stacked bar graph or as pie charts for whichever phylogenetic groupings (e.g., phyla, class, or genera) are to be discussed and/or identified as statistically significant.

The other bioinformatic analyses of interest is the level of microbial diversity present in particular ecosystem or environment. Microbial diversity in essence represents the collective microorganisms that are genetically distinct as defined by 16S rDNA sequencing. Intuitively in any environment the microbial consortia are quite likely to be a mixed conglomerate of individual microorganisms that may or may not be metabolically integrated such as the associations that occur in some GIT systems, particularly the rumen. Likewise, biofilms that can be found in a wide range of environments are likely to consist of multiple microorganisms. Given this complexity, it becomes important to identify the level of complexity both within a particular ecosystem as well as comparisons among ecosystems to assess the impact of external factors such as dietary treatments in animals. Microbiome-based bioinformatics accomplishes this by a variety of diversity metrics. Assessment of taxonomic complexity within a sample or specific environment is determined via an alpha diversity metric, which provides the total number of microorganisms and can be reported as an index number. Comparisons across samples, environments, diets, or different animals, are accomplished using beta diversity estimates that consider the number of shared taxa vs. the taxa that are distinct for each microbial population within these different sources. These comparisons can be presented as Principal Coordinates Analyses (**PCoA**) plots, which provide a 3-dimensional depiction of these relationships and Venn diagrams to illustrate where microbial populations overlap among different sources of interest. Taken together these diversity metrics provide a visualization of how microbial communities compare with each other and illustrate contrasts among their members that comprise these taxonomic groupings.

Unsurprisingly, the food animal industry has begun to embrace microbiome analyses as a potential tool for assessing commercially important factors. Commercial poultry production and processing have also been influenced by the availability of microbiome sequencing and analyses and this has been reviewed in a series of review publications (Feye et al., 2020a,b). In poultry production and nutrition, there is interest in assessing the impact of external factors on the GIT of both broilers and laying hens. For example, the removal of antibiotics in feeds, exposure to stresses such as heat stress and management changes such as shifting egg production to cage-free housing systems have all been the subject of microbiome studies (Feye et al., 2020a; Ricke and Rothrock, Jr., 2020). Microbiome analyses has also been introduced as an analytical tool for commercial poultry processing as well. Based on series of studies summarized by Feye et al. (2020b), the concept of applying microbiome mapping of microbial communities on poultry carcasses as they move through the processing line has been suggested as comprehensive approach to track changes in microbial composition during the various stages of processing. Microbiome mapping can provide an overview of the microbial ecology of poultry processing, but also offers a means for identifying potential indicator microorganisms and/or signature microbial populations. These distinct microbial populations have utility for being reflective of exposure to different processing interventions, as well as potential shelf-life predictors of poultry products.

## APPLICATIONS OF MICROBIOME ANALYSES FOR NON-CONVENTIONAL POULTRY PRODUCTION

Certainly, some of the same issues that impact conventionally raised poultry are also of interest with non-conventional practices, including impacts from the native microbiome. However, there are challenges in the outdoor environments which differentiates some of the potential factors that are more specific to nonconventional poultry production and in some cases may have practical applications beyond the farm. For example, the GIT microbial communities from birds reared under outdoor or free-range settings offers potential sources of microorganisms that may possess unique probiotic properties of commercial value not found elsewhere (Jeni et al. 2021a). Regardless of the initial purpose or potential commercial application, microbiome studies focused on nonconventional poultry have increased in the past few years (Shi et al., 2019; Ricke and Rothrock, Jr., 2020). More recent applications of microbiome analyses have focused on issues related to influential GIT factors on pasture flock production management.

In pasture flock broiler operations, there are number of potentially influential factors that must be considered when assessing the impact on the GIT microbiota. Certainly, environmental stressors such as heat exposure and contact with parasites can contribute to GIT health (Jeni et al 2021b). The opportunity to consume different dietary constituents beyond the conventional corn-soy based diets typically fed conventionally raised birds also likely modulates the GIT microbiota. Free-range broilers have the opportunity to consume different forages depending on the type of pastures they are grazing on during their outdoor foraging. Foraging activities would potentially support a somewhat unique microbiota with the ability to use some fiber fractions. There is precedent for this capability as laying hen studies conducted both in vitro and in vivo have suggested that adult laying hen cecal microbiota can ferment feedstuffs from rations containing high levels of fibers such as alfalfa (Ricke et al., 2013).

Another often overlooked factor that may impact the GIT microbiota is breed of broiler chicken and relative growth period required to reach market weight Montoro-Dasi et al. (2020). compared the cecal microbiomes from fast and slow growing management operations using 2 different commercial breeds of birds. The birds were nutritionally managed under either fast growing conditions with a grow out period of 42 days or a slow growing time frame of 63 days before slaughter. Birds were selected for cecal sampling on day 1, day 21, and on the slaughter date of day 42 for fast growing and day 63 for slow growing broilers. When taxonomic identification was categorized for the differences in breed and management systems, prevalent phyla consisted of Firmicutes followed by Proteobacteria, but Proteobacteria were overtaken by Bacteroidetes during the grow out period. Likewise, *Oscillospira, Ruminococcus, Coprococcus, Lactobacillus*, and *Bacteroides* spp. were the most predominant genera across management systems Montoro-Dasi et al. (2020). concluded that cecal microbiota taxa of birds grown in either management system became stabilized by day 21. When cecal microbial population alpha and beta diversity metrics were generated, age proved to be a significantly influential factor as the alpha diversity complexity index increased with age and the beta diversity comparisons across age were significantly different. In both management systems, day old chicks exhibited the least complex cecal microbiota and this complexity increased at day 21 and again at the endpoint of slaughter.

While the conditions employed by Montoro-Dasi et al. (2020) were not conducted in an outdoor environment, they do indicate that both breed and management system did not markedly influence the cecal microbiota composition. It would be interesting to replicate this study but fully expose the birds to outdoor conditions in an attempt to identify whether housing environments are a factor. In addition, characterizing the cecal contents beyond microbial composition may reveal differences not apparent from a strictly taxonomic analyses basis, and a functional analysis of the end products of fermentation may be more indicative of changes within or between compartments of the GIT (alpha and beta diversity). Finally, application of metagenomic approaches may reveal distinctions at the gene level that can be related to functionality differences in cecal microbial populations present in these different management systems.

The question remains as to the level of influence on GIT microbiota that can be attributable to outdoor environments. To determine this, Schreuder et al. (2021) compared the cloacal microbial communities in laying hens with or without access to an outdoor environment. They used 4 commercial laying hen flocks, all of the same breed, with 2 of the flocks having outdoor access, and 2 flocks housed entirely indoors. All birds were held indoors until the beginning of the study where access to outdoors was granted to the respective flocks. Cloacal swabs were collected from the birds in the first couple of days of outdoor access followed by sampling at 8 and 16 weeks later in the trial. Sequencing of the microbiome 16S rDNA was done on an Illumina MiSeq using variable regions 3 and 4 as the primers and a QIIME R-based analytical software. When cloacal microbial composition was examined at the phyla level, Firmicutes, Proteobacteria, and Fusobacteria were the prevalent taxonomic groups while genera *Romboutsia, Gallibacterium*, and *Fusobacterium* occurred most frequently across all treatments. Any variation in microbial composition detected among the microbial communities was due to housing (9.2 %) and sampling time (4.4%), while outdoor access appeared to have minimal impact on variability in microbial composition. When individual taxa abundance was assessed, the genus *Lactobacillus* varied considerably in outdoor housed birds as did genera *Akkermansia* and *Aeriscardovia,* but not in cloacal samples from indoor held birds. However, the authors pointed out that these were relatively rare taxonomic groups and did not belong to any of the overall predominant taxa. From these results they concluded that the adult cloacal microbiota were relatively resistant to major detectable changes in microbial cloacal composition when birds were suddenly shifted to outdoor settings.

These results are of importance to the pasture flock operator as they support the concept that the potential stress associated with the sudden shifting of birds to an outdoor environment does not appear to substantially alter the GIT microbiota. This would suggest that the adult laying hen GIT microbial community is relatively resilient at this stage of their lifecycle. From a practical management standpoint, this means that the rearing conditions of the young bird may be critical in establishing a beneficial and/or desired GIT microbial consortia, but once established will potentially remain intact (Rothrock, Jr. et al., 2019b). Consequently, the decision of including feed additives such as probiotics and prebiotics or other dietary treatments should be made during the early stages of bird maturity. However, as Schreuder et al. (2021) noted, their results are based on cloacal sampling which may not necessarily be completely representative of the cecal microbial composition. Given the difficulty in invasive sampling approaches, more research needs to be done to develop field acceptable noninvasive sampling approaches to consistently approximate GIT microbial profiles in the bird. This may require large numbers of comparative samples to estimate if consistent differences are sufficiently relative to derive adjustment calculations for the differences that occur.

## UTILITY OF MICROBIOME INFORMATION FOR NONCONVENTIONAL POULTRY PRODUCTION

As more microbiome data is collected for pasture flock operations, it becomes important to appraise the practical utility for management information and subsequent decisions. There are several issues which must be addressed to achieve practical applications for small flock growers. As previously discussed, obtaining representative noninvasive sampling is paramount as the smaller number of birds typically associated with pasture flocks precludes routine sacrificing of birds for experimental purposes. Certainly, removing GIT contents at slaughter can easily be done, but sacrificing birds prior to slaughter is not likely to be supported without economic incentives. However, samples from younger birds are important for evaluation of the impact of feed additives and foodborne pathogen colonization since cecal microbial diversity increases as pasture flock birds mature (Park et al., 2017). To achieve a broader baseline of data will require more studies in general, particularly field studies that are more likely to represent the environmental and other factors that influence performance. This will probably require further development of non-invasive procedures that are more representative of the GIT microbial communities.

Despite the potential difficulties, microbiome data for small flock poultry growers could have value for optimizing management decisions. One application would be deciding which feed additives to use and when to include them in the feed of pasture flock birds. Such decisions could be based on detectable microbiome responses. These responses could allow for the identification of signature GIT microbial populations that provide evidence that a specific feed additive is functioning in the GIT as expected. For example, Park et al. (2017) identified statistically significant increases in certain members of the cecal population when prebiotics were included in the diets of 6-week old pasture flock birds. Likewise, when Naked Neck pasture flocks were fed a commercial yeast cell wall product, detectable increases in certain members of the cecal microbial population were detected (Park et al., 2016). As more microbiome data is generated using pasture flock birds, the development of specific management tools for not only assessing the impact of including feed supplements but developing more optimal dosages and determining which feed additives to use for a specific small flock operation can be realized and recommendations made.

The opportunity to provide scientific evidence for making decisions such as major dietary changes may become possible. For example choosing between soy-free vs. soy-based diets in broiler pasture flock and laying hen cage free operations (Al-Ajeeli et al., 2018; Lourenco et al., 2019a,b). Such information could help with deciding when to make the shift to retain GIT health, minimize stress, and achieve optimal performance. Likewise, management decisions on when to release birds to outdoor environments, when to move a pen of birds in the pasture, or initiate flock rotation with other animal species could be based on microbiome data. Such data could be used to identify biomarker or indicator GIT microbial populations detectable via noninvasive procedures such as fecal sampling or via cloacal swabs for on-site tests. In addition, establishing a large database may preclude the need for further sampling as there may be sufficient data to derive mathematical models for generating standard operating procedures and decision tree guidance of when to make management decisions. Predictive modeling could also be used to connect bioinformatic information with readily measurable metrics such as average minimum temperature or other environmentally collectable data that could be used in predictor equations for assessing issues such as pathogen exposure risk or GIT health.

However, to achieve routine management tools utilizing molecular methods will require further development and optimization of assays and sampling procedures. Part of the difficulty of implementation lies in the fact that because these birds are free ranging; simply getting access to the birds in a timely fashion is a challenge. However, new technologies such as the smart robot system being proposed for visual guidance of egg collection, global positioning systems, and radio frequency identification (RFI) to track individual birds (Dal Bosco et al., 2010; Larsen et al. 2017; Chang et al., 2020) might provide opportunities to combine these technologies with relevant types of sample collections for monitoring grazing birds. While some of these technologies may not be completely adaptable, further investigation is warranted if viable sampling is to occur in the grazing environment settings that these birds are raised. In addition, GIT microbial community differences in management systems, environmental differences, bird breed and age, as well as host responses such as the immune system will need to be delineated. Finally, development of education materials that can effectively communicate the seemingly complex bioinformatics information to a small poultry producer audience will be critical for widespread acceptance and application of this type of information.

## AGRICULTURAL EDUCATION AND NONCONVENTIONAL POULTRY PRODUCERS

Historically, agricultural education has been an important cornerstone of higher education institutions in the United States with the introduction of the land grant university system (Ghimire et al., 2014). However, the agriculture landscape is rapidly changing, presenting new challenges for farm practitioners as well as the agricultural institutions. There are several factors that directly or indirectly impact overall status of agricultural practices and policies and the educational materials that might be required; for example, how effectively legislators use scientific evidence for implementing governmental policy (Crowley et al., 2021). This can have potential impact on regulatory policy of the practices that are considered by the government to be defined as natural or organic agriculture vs. what is considered conventional agricultural operations. Regulatory policies regarding environmental management of agricultural land, food labeling, food safety, and meat processing inspection, among others can all be factors in livestock operations. Consequently, agricultural curricula that includes coursework on food law, food safety, animal welfare, and environmental sustainability become more important as agricultural college graduates enter the workforce.

The other challenge that agriculture faces is the technological evolution that is overtaking agricultural management practices. Rose and Chivers (2018) have described this as “smart farming” which employs several technological advances such as the use of drones for mapping crop land, robots for performing certain menial tasks, and extensive use of digital data sets and programming. This technological evolution is driving intensification of sustainable agriculture and is influencing research as well as policy. For example, Esnaola-Gonzalez et al. (2020) developed a cloud-based IoT (Internet of Things) platform for poultry production chains designed to collect data at all stages to determine where efficiency could be improved. The concept of “big data” is also impacting the retail food industry, which is turn impacts the agriculture sector through food safety and the need for traceability technologies (Strawn et al., 2015). While many of these technological advances may seem remote to a small flock poultry grower, the increased sophistication of their customers with enhanced smart phone technologies that allow identification of the sources of their food purchases will have an impact. To illustrate this, Chen et al. (2017) examined repurposing barcodes on packaging materials as colorimetric sensor array images to reflect the quality status of chicken breasts stored in the refrigerator that can be captured via a smart phone. As these types of technologies develop, it is conceivable that other information can be collected on retail chicken products such as farm source, management practices, and a record of food safety. As the demand for organic and pasture-raised poultry increases, this technology will likely become a means for accountability by the consumer on the authenticity of origin of retail poultry that will, in turn, require more data inputs throughout the poultry production chain.

Given the rapid penetration of advanced technologies into agricultural practices, educational materials need to be developed to address the needs of small flock poultry growers in the context of rapid technological advances being made throughout agriculture. A key part of communication and delivery of educational materials is understanding the background, interests, and motivations of the audience. While the motivations for pasture flock growers to raise chickens on free range operations have been identified in several studies (Ricke and Rothrock, Jr. 2020), much less in known about their demographics. Elkhoraibi et al. (2014) conducted a national online survey of backyard flock owners who were at least 18 years old and raised between 1 and 50 birds. Backyard producer respondents were evenly distributed (approximately 33%) among urban, suburban, and rural locations, and nearly a third had at least a 4-year college degree and over a third more possessed a graduate or professional degree with the majority of the producers being female. As the authors point out, this particular survey likely underrepresents other demographic groups also involved in backyard chicken producers since it was disproportionately represented by California respondents. Unsurprisingly, over three fourths of the backyard flock growers chose to use websites, followed by email newsletters, and finally in-person workshops for communication. All of these communication forums represent opportunities to deliver educational material of different types such as lectures online vs. demonstrations in onsite attended workshops.

For educating producers on the use of advanced technologies such as microbiome analyses, both types of forums may be useful depending on the content and applications being discussed. However, before such materials are developed, further demographic information must be generated from a much broader set of demographic surveys to better capture the educational backgrounds of small poultry flock growers. Not only does this type of information require interpretation in an understandable language for the lay person, but the individuals delivering the educational content also require training to develop the educational materials and deliver them effectively to an audience with a wide range of educational backgrounds. Based on a systematic assessment of higher educational institutions, Benaivides et al. (2020) concluded that digital transformation has permeated higher education institutional operations in several ways. They reported that digital transformations were occurring at multiple levels of development of digital platforms for teaching infrastructure, curricula modernization, and administration among others. While it might be assumed that incoming college students are somewhat aware of digital technologies, how extensive their background is unclear. In a survey of undergraduate and graduate students interested in food safety, Feye et al. (2020c) concluded that students were aware of some digital venues such as social media and word processing software, but much less so for digital technologies associated with web design and cybersecurity. Not surprisingly, Martins et al. (2020) observed similar lack of familiarity in high school students with the terminology and definition of bioinformatics. To remedy these deficiencies, Feye et al. (2020c) suggested developing more cross disciplinary curricula that combine elements of practical agriculture sciences with computer and cybersecurity topics. These interfaces will become particularly important for adaptation of monitoring and data gathering based on microbiome bioinformatic interpretations by industries such as those with poultry processing facilities (Feye et al., 2020b,c).

If technologies based on microbiome sequencing and bioinformatics are to become a staple in agricultural sciences, they will need to become more than just an academic research exercise. Educational and extension activities must be developed to provide the appropriate training and information sources for students and the public. This is particularly true for students planning to enter the agricultural workforce where digital data gathering for monitoring purposes has become commonplace. Recruiting students into agricultural careers such as poultry production remains a challenge given the limited number of universities offering a comprehensive poultry science curriculum, coupled with the minimal exposure of students to agriculture prior to entering college (Wickenhauser et al., 2021). In addition, university extension services are attempting to balance between interactions with the major poultry industries, and still meet the needs of small flock producers such as those involved in pasture flock operations (Pohl et al., 2010). Finally, educational and extension materials that provide background on newer technologies such as microbiome sequencing and bioinformatic interpretations from a practical standpoint need to be developed, including providing the technical background as well as constructing the educational materials in a user-friendly format for a multitude of lay audiences.

## EDUCATIONAL APPROACHES FOR COMMUNICATING MICROBIOME SEQUENCING AND BIOINFORMATICS

Communicating information gleaned from microbiome sequencing and bioinformatics analyses to the highly varied educational backgrounds of a small flock poultry grower audience represents several challenges; however, this is only part of a more fundamental problem in public education. In general, the genetic and genomic technology literacy of the public remains limited despite the introduction of concepts such as personalized medicine (Sassano et al., 2021; Zimani et al., 2021). In the survey conducted by Sassano et al. (2021), they identified a considerable heterogeneity in delivering information on “omics” sciences to the public with both in-person and web-based sources being utilized. Topics made available in these forums included: cellular biology basic concepts, genetics, genetic disease risks along with modern genomic sequencing methods, genetic tests, and related subjects. In-person information was delivered via exhibitions, seminars, courses, symposia, research laboratory tours, as well as interactive laboratory exercises.

Certainly, delivering information on microbiome and bioinformatic technologies to both small flock growers as well as their perspective customers will likely require a range of different forums. Depending on the need, in-person workshops for some producers might be optimal while a large portion of background content could probably be more effectively delivered via online programs. Both basic concepts as well as more sophisticated technical material could be delivered via either forum; however, hands-on training for sequencing and generating raw data may be necessary to gain an appreciation of the complexity of some of the steps and the rationale for the laboratory approaches taken. For example, the importance of utilizing particular protocols for isolating DNA from different matrices that results in an optimal yield and quantity for sequencing may need in-person instruction to gain appreciation on what is required for field sampling. This may be especially necessary if field samples are to be collected and shipped to a centralized laboratory location. Other components such as basic concepts and background for the 16S rDNA basis of microbiome sequencing can probably be delivered in some form of lecture or webinar online. Bioinformatics training may require both workshop and hands-on training as well as interactive web-based exercises that provide example data sets or the opportunity for the trainee to work with their own microbiome raw sequence data.

Increased efforts to develop educational materials and laboratory exercises for microbiome training at both the high school and college undergraduate level may change the level of familiarity with microbiome/bioinformatics in the next generation. Muth and Caplan (2020) summarized the efforts in different universities to develop microbiome research projects for undergraduates. Based on their surveys of biology faculty, they concluded that for some aspects such as quantitative and data processing, the lack of basic computing background in students, insufficient bioinformatic lesson plans, coupled with the rapidly changing bioinformatic program content were challenges along with limited faculty expertise and time. The idea of cross training between the disciplines of computer science and other fields such as food science may offer a partial solution to some of this by creating interactions between the 2 groups during classroom discussions (Feye et al., 2020c). This is also consistent with Muth and Caplan's (2020) observation that microbiome projects support development of critical thinking, problem solving, and collaboration skills which, in turn is more reflective of the changing requirements of the workforce. This holds true for food safety issues in the poultry food industries as well (Dittmar et al., 2006; Ricke, 2015a; Thompson et al., 2017). As Muth and Caplan (2020) suggest, the teaching elements involved in undergraduate microbiome research projects offer insights into topics of concern to the public sector such as climate change, food security, and human health.

Martins et al. (2020) designed 4 bioinformatics courses for high school biology classes that covered genomic concepts and genomic data mining. They selected a genomic region of *Escherichia coli* to base their bioinformatic exercises on and, in turn determine its appearance in other bacterial taxa. Bioinformatic resources used included Open Reading Frames Finder (ORFfinder) and Basic Local Alignment Search Tool (**BLAST**), among others and a webpage was developed specifically for teachers to provide bioinformatic instructional materials. Martins et al. (2020) outlined the steps for delivering these exercises to the students and included initial genomic background presentation, followed by introduction to bioinformatic databases, then execution of the particular bioinformatics activity, and as a final step of implementation where results were discussed, and conclusions reached. When Martins et al. (2020) surveyed students after completing this exercise, they could identify bioinformatic tools and better handle the misconceptions associated with genomics. Based on these results, Martins et al. (2020) suggested that these types of exercises could be developed for younger audiences.

Development of data processing platforms have also been explored in an attempt to make microbiome analytics more user friendly for a broad audience. Mitchell et al. (2020) noted the difficulty of beginners to grasp the microbiome data processing systems that require command line interfaces. Even when graphical user interface capabilities are available for visualization, thus making it easier, Mitchell et al. (2020) emphasized the universal data formats still do not exist which would allow integration. Consequently, microbiome analytics are still challenging for inexperienced users. This includes undergraduate college students who are being introduced to microbiome topics as part of their curricula. In response to this educational void, Mitchell et al. (2020) developed an approach entitled Program for Unifying Microbiome Analysis Applications (**PUMAA**). As a part of PUMAA, formatting of raw data files to graphical user interface programs occurs that generates visual presentations of taxonomy and predicted functionality. This, in combination with tutorials, allowed students without computational background to successfully complete microbiome bioinformatics analyses. To develop more understanding on computational tools, Kruchten (2020) used YouTube videos to provide faculty training on metagenomics and R analyses for delivering a wet lab and an *in silico* undergraduate research experience presented as a course held for several weeks.

## CONCLUSIONS

Poultry growers involved in free-range and cage-free egg production are continuing to provide poultry products that are sought after by consumers. This is in part due to the interest in locally produced agriculture products as well as other perceived benefits associated with these types of products. However, as market demand continues to grow, more sophisticated management tools will be needed to accommodate these increases. There are several reasons for this. First of all, there are management challenges to sustain a healthy flock due to more exposure to environmental extremes of outdoor production and the limited availability of health promoting feed additives deemed acceptable to this type of production. There are nutritional hurdles as well, not only because of the varied choices in feed components, but the opportunity by free ranging birds to consume forages, insects, and miscellaneous substances they encounter while grazing. Finally, the need to anticipate shifts in market demands and the expectations of potential customers can lead to a requirement for relatively fluid management decisions such as where and when to process broilers.

The introduction of microbiome sequencing technology to the animal industry is no longer novel but is becoming a more acceptable analytical method as more data is generated. The sequencing technology is evolving and thus becoming more cost effective. This has promoted more routine data interpretations and a wider range of applications. While there has been considerable research conducted with conventional animal and poultry production systems, some work has also been generated from non-conventional animal operations including pasture flock and cage-free produced birds. The information generated from these studies have provided insights into a number of issues of interest to management such as impacts on the GIT microbiota during changes in diets, rotation of pens throughout the pasture, food safety, and exposure to different stresses. While these represent promising opportunities for potential applications of microbiome data, interpreting and delivering the information in educational formats that are user friendly for small flock poultry grower audiences is a hurdle that must be overcome if these approaches are to be practical.

Educating the small flock poultry growers on the technical aspects of microbiome analyses may seem challenging, but inroads are being made to make this easier to accomplish. Several factors are contributing to this transition. First of all, the need and practical applications for microbiome analyses is becoming more apparent as a useful diagnostic tool to animal agriculture. This trend will continue as more data is generated from both controlled experiments as well as samples collected from field studies. Secondly, as microbiome technology becomes more popular, educational tools for effectively delivering the concepts and methods for sequencing and bioinformatics are expanding with new and innovative forums such as videos, webinars, and interactive internet programming to deliver content. In addition, computer programs that are more user friendly are being developed for meeting the needs of students with either minimal or no computational skills. This will have both a direct and indirect impact on small flock poultry growers. A direct impact will be that these same educational tools can be used for delivery of user-friendly educational materials to small flock poultry growers both for online instruction as well as in-person workshops. While not as obvious, there is an indirect impact as well. As these students leave high school and college and enter the workforce their understanding of new technologies related to genomics and bioinformatics will be greater than their predecessors due to the increased efforts to introduce these materials into the curricula. These former students will also be potential customers of pasture flock raised poultry products and will have more appreciation for this type of information and likely request trackable data as part of their demand for accountability on the origins and sources of the food they purchase.

## DISCLOSURES

There is no conflict of interest for any of the authors.
